# Supplementary material for: Prevalence and associated factors of acute respiratory infection among street sweepers and door-to-door waste collectors in Dessie City, Ethiopia: A comparative cross-sectional study
Source: PLoS One. 2021 May 14;16(5):e0251621. doi: 10.1371/journal.pone.0251621 (PMC8121341; doi:10.1371/journal.pone.0251621)
Supplement: S1 Questionnaire — Survey of prevalence and associated factors of acute respiratory infection among street sweepers and door-to-door waste collectors in Dessie City, Ethiopia. (DOCX) [file pone.0251621.s001.docx]

## Questionnaire (English version)

**Wollo University**

**College of medicine and health science Department of environmental health**

Date of interview………………………

Questionnaires number …………

Name of interviewer……………………

Please, put check (√) in front of the correct question number which to indicate number and write a correct number example age put in a year

|  | Socio-demographic Information |  | response |
| --- | --- | --- | --- |
| s.no | Question | Coding classification |  |
| 1.10 | Occupation | 1 = street sweepers |  |
|  |  | 2= door-to-door waste collectors |  |
| 1.11 | Sex | 0 = Male |  |
|  |  | 1 = female |  |
| 1.12 | Age ……………………………………………. Years | |  |
| 1.13 | Marital status: Marriage | 1 = Single |  |
|  |  | 2 = Married |  |
|  |  | 3= Separated |  |
|  |  | 4 = Widow |  |
| 1.14 | Level of education | ------------------class |  |
| 1.15 | How long in this job? | ……………….years |  |
| 1.16 | How many working hours per day? | -------------hours |  |
| 1.17 | How many working hours per a week? | ----------------hours |  |
| 1.18 | Number of family members ----------------------- | | |
|  | What is your income per month?-------------------birr | | |
| 1.20 | What is your household income per month?--------------------birr | | |

**II. Behavioral factors**

| S.NO | **Questions** | | **Respond** |
| --- | --- | --- | --- |
| 2.10 | Do you smoke cigarette? | 0 = No |  |
|  |  | 1= Yes |  |
| 2.11 | If you say yes for question 1 when did you begin to smoke? | 1=Before I started this work |  |
|  |  | 2=After I started this work |  |
| 2.12 | Do you drink alcohol? | 0=No |  |
|  |  | 1=Yes |  |
| 2.13 | If you say yes for question 3 when did you start? | 1=Before I started this work |  |
|  |  | 2=After I started this work |  |
| 2.14 | Do you chew chat? | 0=No |  |
|  |  | 1=Yes |  |
| 2.15 | If you say yes for question 5 when did you begin to chew? | 1=Before I started this work |  |
|  |  | 2=After I started this work |  |

**III. PAST RESPIRATORY DISEASES HISTORY**

| **Questions** | **Respond** | | When In year ,month |
| --- | --- | --- | --- |
| Was either of your past life ever told by a doctor that you had a chronic lung condition as mentioned below? | 0 = No, | 1 = Yes |  |
| 3.10 Chronic bronchitis |  |  |  |
| 3.12Emphysema |  |  |  |
| 3.14 Asthma |  |  |  |
| 3.16 Heart attack. |  |  |  |
| 3.18 Lung cancer. |  |  |  |
| 3.20 pulmonary Tuberculosis (TB) |  |  |  |
| 3.22Pneumonia |  |  |  |
| 3.24Sinus |  |  |  |

**IV. AVAILABILITY OF PPES**

| **S/N** | **Type of PPEs** | **Availability of PPEs** | | |
| --- | --- | --- | --- | --- |
|  |  | **Answer** | | |
|  |  | **No = 0** | **Yes = 1** | **Explanations (condition of PPE)** |
| 4.10 | Do you have PPEs |  |  |  |
| **If No go to part VI** | | | | |
| 4.11 | Nose/mouth masks |  |  |  |
| 4.12 | Hand gloves |  |  |  |
| 4.13 | Eye protection /goggles |  |  |  |
| 4.14 | Foot wear (boots) |  |  |  |
| 4.15 | Clothing/apron/overall |  |  |  |
| 4.16 | Reflectors |  |  |  |
| 4.17 | Helmet |  |  |  |
| 4.18 | Availability of shower |  |  |  |
| 4.19 | Where did you get PPEs---------------------------------------------------- | | | |
| 4.20 | Do you clean your PPE while using it. |  |  |  |
| 4.21 | Do you take a shower after work |  |  |  |

**V. TRAININGS**

| S.NO | Question | | | Code | Respond | | |
| --- | --- | --- | --- | --- | --- | --- | --- |
| 5.10 | Have you ever trained about the importance of  Personal protective device and their application? | | No=0 | | |  | |
|  |  |  | Yes=1 | | |  | |
| 5.11 | If yes, who gave you the training | | 1. Health professional | | |  | |
|  |  |  | 1. Other (specify-----) | | |  | |
| If your answer is yes for question number 1 | | | | | | | |
| 5.12 | When did you trained? | 1=Before you start this job | | | | |  |
|  |  | 2=After you start this job | | | | |  |
| 5.13 | When------------------------------------month | | | | | |  |

**VI: OCCUPATIONAL HISTORY**

| S/N | Questions | Coding classification | Respond |
| --- | --- | --- | --- |
| 6.10 | Have you ever worked for any other dusty job? | 0 = No |  |
|  |  | 1= Yes |  |
| 6.11 | When-------------------------------------------year | |  |
| 6.12 | Have you ever been exposed to gas or chemical fumes in any other work? | 0 = No |  |
|  |  | 1= Yes |  |
| 6.13 | When-------------------------------------------year | |  |

**VII. ARI SYMPTOMS**

| S.NO | Question | Coding | Respond |
| --- | --- | --- | --- |
| Have you ever experienced the following ARI symptoms listed down below in the preceding two weeks | | | |
| 7.10 | Runny nose | 0= No |  |
|  |  | 1= Yes |  |
| 7.11 | Cough | 0= No |  |
|  |  | 1= Yes |  |
| 7.12 | Fever | 0= No |  |
|  |  | 1= Yes |  |
| 7.13 | Sore throat | 0= No |  |
|  |  | 1= Yes |  |
| 7.14 | Chest tightness | 0= No |  |
|  |  | 1= Yes |  |
| 7.15 | Ear discharge | 0= No |  |
|  |  | 1= Yes |  |
| 7.16 | Wheezing | 0= No |  |
|  |  | 1= Yes |  |
| 7.17 | Is there any symptom of ARI listed above | 0= No |  |
|  |  | 1= Yes |  |

**Thank you very much once again for your participation!!**

## Checklist

**I. UTILIZATION OF PPEs**

| **S/N** | **Type of PPEs** | **Wearing of PPEs during working (observational)** | | |
| --- | --- | --- | --- | --- |
|  | **Answer** | | | |
|  |  | **No = 0** | **Yes= 1** | **Explanations** |
| 1.10 | nose/mouth mask |  |  |  |
| 1.11 | Hand gloves |  |  |  |
| 1.12 | Eye protection /goggles |  |  |  |
| 1.13 | Foot wear (boots) |  |  |  |
| 1.14 | Clothing/apron/overall |  |  |  |
| 1.15 | Reflectors |  |  |  |
| 1.16 | Helmet |  |  |  |

**II. HOUSING CONDITION**

| **S.NO** | Question | | | Code | | | Respond |
| --- | --- | --- | --- | --- | --- | --- | --- |
| 2.10 | | Type of energy used at home…………………………….. | | | | | |
| 2.11 | | What type of Building material is used to construct the house? | | | 1=Cement | |  |
|  |  |  |  |  | 2=Mud | |  |
| 2.12 | | Floor material of the house | | | 1=Tile/ceramic | |  |
|  |  |  |  |  | 2=Soil | |  |
| 2.13 | | Kitchen location | | | 1=Separate | |  |
|  |  |  |  |  | 2= Not separate | |  |
| 2.14 | | Does the bedroom has a window | | | 0=No | |  |
|  |  |  |  |  | 1= Yes | |  |
| 2.15 | | Is there pets (dog or cat) in the house | | | 0=No | |  |
|  |  |  |  |  | 1= Yes | |  |
| 2.16 | | Is there dove or chicken or farm animal in the house | | | 0=No | |  |
|  |  |  |  |  | 1= Yes | |  |
| 2.17 | | Number of person per room is (asking)…………………………. | | | | | |
| 2.18 | | Cigarette smoker in house(asking) | 0=No | | |  | |
|  |  |  | 1= Yes | | |  | |

**Thank you very much once again for your participation!!**
