# Supplementary material for: Prevalence and associated factors of acute respiratory infection among street sweepers and door-to-door waste collectors in Dessie City, Ethiopia: A comparative cross-sectional study
Source: PLoS One. 2021 May 14;16(5):e0251621. doi: 10.1371/journal.pone.0251621 (PMC8121341; doi:10.1371/journal.pone.0251621)
Supplement: S2 Questionnaire — Survey of prevalence and associated factors of acute respiratory infection among street sweepers and door-to-door waste collectors in Dessie City, Ethiopia. (DOCX) [file pone.0251621.s002.docx]

## Questionnaire (Amharic version)

የመረጃመጠየቂያቅጽ

ወሎ ዩኒቨርሲቲ

ህክምናና ጤና ሳይንስ ኮሌጅ የአካባቢ ጤና አጠባበቅ ትምህርት ክፍል

ቃለመጠይቅ የተደረገበት ቀን………………………

የመጠይቅ ቁጥር…………

የጠያቂው ስም……………………

በትክክለኛው የጥያቄ ተራ ቁጥር ከምላሹ ፊት ለፊት ባለው ምላሽ መስጫ ላይ የምላሹን ኮድ ያስቀምጡ ወይም በተስጠዉ ክፍት ቦታ ላይ ለምሳሌ እድሜ ትክክለኛውን ቁጥር ያስቀምጡ

| ተ.ቁ | ጥያቄ | ኮድ | ምላሽ |
| --- | --- | --- | --- |
| 1.10 | የስራ ሁኔታ | 1 = የመንገድ ፅዳተኛ |  |
|  |  | 2= የቤትለቤት ደረቅ ቆሻሻ አስወጋጅ |  |
| 1.11 | ፆታ | 0 = ወንድ |  |
|  |  | 1 = ሴት |  |
| 1.12 | ዕድሜ______________________________________ዓመት | |  |
| 1.13 | የጋብቻሁኔታ | 1 = ያላገባ(ች) |  |
|  |  | 2 =ያገባ(ች |  |
|  |  | 3= የፈታ(ች) |  |
|  |  | 4 =የሞተበት(ባት) |  |
| 1.14 | የትምህርት ደረጃዎ | 1 =ማንበብና መፃፍ የማይችል |  |
|  |  | 2 = ማንበብና መፃፍ የሚችል |  |
|  |  | 3 = የመጀመሪያ ደረጃ |  |
|  |  | 4= ሁለተኛ ደረጃ |  |
|  |  | 5 = ዲፕሎማና ከዚያ በላይ |  |

| 1.15 | ይህንን ስራ መስራት ከጀመሩ ስንት ጊዜ ይሆንዎታል ………………………..አመት |
| --- | --- |
| 1.16 | በቀን ምን ያህል የስራ ሰዓታት ይስራሉ? ………………………ሰዓት |
| 1.17 | በሳምንትምን ያህል የስራ ሰዓታት ይስራሉ? ………………………..ሰዓት |
| 1.18 | የቤተሰብ አባላት ብዛት…………………………….. |
| 1.19 | የወርገቢዎ ስንት ነው ……………………………..ብር |
| 1.2 | ጠቅላላ የቤተሰብዎ የወርገቢ ስንት ነው……………..ብር |

**ክፍል 2. የባህሪ ሁኔታ**

| ተ.ቁ | ጥያቄ | | ምላሽ |
| --- | --- | --- | --- |
| 2.10 | ሲጋራያጨሳሉ? | 0 = አይ |  |
|  |  | 1 = አዎን |  |
| 2.11 | መልስዎ አዎ ከሆነ መቼ ማጨስ ጀመሩ? | 1= ይህን ስራ ከመጀመሬ በፊት |  |
|  |  | 2=ይህን ስራ ከመጀመሬ በኃላ |  |
| 2.12 | የአልኮል መጠጥ ይጠጣሉ? | 0 = አይ |  |
|  |  | 1 = አዎን |  |
| 2.13 | ለጥያቄ ቁጥር 3 መልስዎ አዎ ከሆነ መቼ መጠጣት ጀመሩ? | 1= ይህን ስራ ከመጀመሬ በፊት |  |
|  |  | 2=ይህን ስራ ከመጀመሬ በኃላ |  |
| 2.14 | ጫት ይቅማሉ? | 0 = አይ |  |
|  |  | 1 = አዎን |  |
| 2.15 | ለጥያቄ ቁጥር 5 መልስዎ አዎ ከሆነ መቼ መቃም ጀመሩ? | 1= ይህን ስራ ከመጀመሬ በፊት |  |
|  |  | 2=ይህን ስራ ከመጀመሬ በኃላ |  |

**ክፍል 3. የቀደመየጤናችግር**

| ጥያቄ | ምላሽ | |
| --- | --- | --- |
| ከታች ከተገለጹት በሃኪም የተረጋገጠ የጤና ችግር አለብዎት/ነበረብዎ | 0 = አይ | 1 = አዎ |
| 3.10 የጉሮሮ ቁስለት (ብሮንካይትስ) |  |  |
| 3.11 መልስዎ አዎ ከሆነ መቼ (በአመት ወይም በወር -------------------------------------------- | | |
| 3.12 ለመተንፈስ የሚያውክ የሳንባ በሽታ ( ኢምፋይሴማ) |  |  |
| 3.13 መልስዎ አዎ ከሆነ መቼ (በአመት ወይም በወር) -------------------------------------- | | |
| 3.14 አስም |  |  |
| 3.15 መልስዎ አዎ ከሆነ መቼ (በአመት ወይም በወር) -------------------------------------- | | |
|  |  |  |
| 3.16 የልብ ህመም |  |  |
| 3.17መልስዎ አዎ ከሆነ መቼ (በአመት ወይም በወር) -------------------------------------- | | |
| 3.18 የሳንባ ካንሰር |  |  |
| 3.19 መልስዎ አዎ ከሆነ መቼ (በአመት ወይም በወር) -------------------------------------- | | |
| 3.20 የመተንፈሻ አካል ነቀርሳ(ሳንባ ነቀርሳ ) |  |  |
| 3.21 መልስዎ አዎ ከሆነ መቼ (በአመት ወይም በወር) -------------------------------------- | | |
| 3.22 የሳንባ ምች |  |  |
| 3.23 መልስዎ አዎ ከሆነ መቼ (በአመት ወይም በወር) -------------------------------------- | | |
| 3.24 ሳይነስ |  |  |
| 3.25መልስዎ አዎ ከሆነ መቼ (በአመት ወይም በወር) -------------------------------------- | | |

**ክፍል 4. ራስን ከኣደጋ ለመከላከል የሚያስችሉ መገልገያ መሳሪያዎችን የማግኝት ዕደል**

| ተ.ቁ | ጥያቄ | ምላሽ | | |
| --- | --- | --- | --- | --- |
|  |  | አይ= 0 | አዎ= 1 | ማብራሪያ |
| 4.10 | የግል የኣደጋ መከላከያ መገልገያ መሳሪያዎች አለዎ |  |  |  |
|  | መልስዎ አዎ ከሆነ | | |  |
| 4.11 | የአፍና አፍንጫ መሸፈኛ |  |  |  |
| 4.12 | የእጅ ጓንት |  |  |  |
| 4.13 | የአይን መከላከያ/ጉግል |  |  |  |
| 4.14 | ጫማ/ቡት |  |  |  |
| 4.15 | ልብስ/ ሽርጥ |  |  |  |
| 4.16 | የጸሀይ መከላከያ |  |  |  |
| 4.17 | የአደጋ መከላከያ ኮፍያ |  |  |  |
| 4.18 | የገላ መታጠቢያ አገልግሎት ከድርጅታችሁ ታገኛላች? |  |  |  |
| 4.19 | የግል የኣደጋ መከላከያ መገልገያ መሳሪያዎቹን ከየት አገኙ |  |  |  |
| 4.20 | ግልየኣደጋ መከላከያመገልገያ መሳሪያዎቹን ከስራ በሃላ ያጸዳሉ |  |  |  |

**ክፍል 5. ስልጠናን በተመለከተ**

| ተ. ቁ | ጥያቄ | | ኮድ | | ምላሽ | | |
| --- | --- | --- | --- | --- | --- | --- | --- |
| 5.10 | ስለ የግል የኣደጋመከላከያመገልገያ መሳሪያዎች ጥቅምና አጠቃቀም ስልጠና ወስደው ያውቃሉ? | | | 0=አይ | |  | |
|  |  |  |  | 1=አዎ | |  | |
| 5.11 | መልሰዎ አዎ ከሆነ ስለጠናዉ በማን ተሰጠዎት | | | 1.በጤና ባለሙያ | |  | |
|  |  |  |  | 2.ሌላ(ይገለጽ) | |  | |
| 5ጥያቄ 1መልስዎ አዎ ከሆነ | | | | | | | |
| 5.12 | ስልጠናውን መቼ ወሰዱ? | 1=ስራውን ከመጀመርዎ በፊት | | | | |  |
|  |  | 2=ስራውን ከጀመሩ በኋላ | | | | |  |
| 5.13 | መቼ | ………አመት | | | | |  |

**ክፍል 6፡ የቀደመ የስራ ሁኔታ**

| ተ.ቁ | ጥያቄ | ኮድ | ምላሽ |
| --- | --- | --- | --- |
| 6.10 | ሌላ ለአቧራ የሚያጋል ጥስራሰርተዉያዉቃሉ? | 0 = አይ |  |
|  |  | 1= አዎ |  |
| 6.11 | መቼ……… ………………. .አመት/ወር | | |
| 6.12 | ሌላ ለመርዛማ ጭስ ወይም ለኬሚካል ጋዝ የሚያጋልጥ የስራ ቦታ ሰርተዉ ያዉቃሉ? | 0 = አይ |  |
|  |  | 1= አዎ |  |
| 6.13 | መቼ……… ………………. .አመት/ወር | |  |

**ክፍል 7: የመተንፈሻ አካል ህመም ምልክቶች**

| ተ.ቁ | ጥያቄ | ኮድ | ምላሽ |
| --- | --- | --- | --- |
| በዚህ ሁለት ሳምንት ጊዜ ዉሰጥ ከታች የተዘረዘሩት የትኞቹ የመተንፈሻ አካል ህመም ምልክቶች ታይቶብዎታል | | | |
| 7.10 | የአፍንጫ ፈሳሽ(ንፍጥ) መዝርክረክ | 0=አይ |  |
|  |  | 1= አዎ |  |
| 7.11 | ሳል | 0=አይ |  |
|  |  | 1= አዎ |  |
| 7.12 | ትኩሳት | 0=አይ |  |
|  |  | 1= አዎ |  |
| 7.13 | የጉረሮ መድረቅ ወይም ማሳከክ | 0=አይ |  |
|  |  | 1= አዎ |  |
| 7.14 | የደረት ውጋት | 0=አይ |  |
|  |  | 1= አዎ |  |
| 7.15 | የጆሮ ፈሳሽ መግል ወይም ደም | 0=አይ |  |
|  |  | 1= አዎ |  |
| 7.16 | ደረት ላይ ሲርሲ የሚልድምጽ | 0=አይ |  |
|  |  | 1= አዎ |  |
| 7.17 | ከላይ ከተጠቀሱት አንዱ የመተንፈሻ አካል ህመም ምልክቶች ተከስቷል | 0=አይ |  |
|  |  | 1= አዎ |  |

ስለተሳትፎዎ እናመሰግናለን !!

## Checklist (Amharic version)

**ክፍል 1. ራስን ከኣደጋ ለመከላከል የሚያስችሉ መገልገያ መሳሪያዎችን መጠቀም**

| ተ.ቁ | መረጃ በሚሰበሰብበት ጊዜ የትኞቹን የግል የኣደጋ መከላከያ መገልገያ መሳሪያዎች ተጠቅመዋል | ምላሽ | | |
| --- | --- | --- | --- | --- |
|  |  | አይ= 0 | አዎ= 1 | ማብራሪያ |
| 1.10 | የአፍና አፍንጫ መሸፈኛ |  |  |  |
| 1.11 | የእጅ ጓንት |  |  |  |
| 1.12 | የአይን መከላከያ/ጉግል |  |  |  |
| 1.13 | ጫማ/ቡት |  |  |  |
| 1.14 | ልብስ/ ሽርጥ |  |  |  |
| 1.15 | የጸሀይ መከላከያ |  |  |  |
| 1.16 | የአደጋ መከላከያ ኮፍያ |  |  |  |
| 1.17 | ከስራ በኃላ ገላዎን ይታጠባሉ |  |  |  |

**ክፍል 2. የቤት ሁኔታ**

| **ተ.ቁ** | ጥያቄ | | | ኮድ | | | ምላሽ |
| --- | --- | --- | --- | --- | --- | --- | --- |
| 6.10 | | በቤት ውስጥ የሚጠቀሙት የማብሰያ ሃይል ምንጭ ምንድን ነው?..................................... | | | | | |
| 6.11 | | የመኖርያ ቤቱ የተገነባበት ማተሪያል? | | | 1=ሲሚንቶ | |  |
|  |  |  |  |  | 2=ጭቃ | |  |
| 6.12 | | የመኖርያ ቤቱ ወለል ማተሪያል | | | 1=ምንጣፍ/ሴራሚክ | |  |
|  |  |  |  |  | 2=አፈር | |  |
| 6.13 | | ኩሽና ከመኖርያ ቤቱ | | | 1=የተለየ | |  |
|  |  |  |  |  | 2= ያልተለየ | |  |
| 6.14 | | የመኝታ ቤቱ መስኮት አለው? | | | 0=አይ | |  |
|  |  |  |  |  | 1=አዎ | |  |
| 6.15 | | በመኖርያ ቤቱ ለማዳ የቤት እንስሳ(ዉሻ ወይም ድመት) አለ? | | | 0=አይ | |  |
|  |  |  |  |  | 1=አዎ | |  |
| 6.16 | | በመኖርያ ቤቱ ውስጥ እርግብ ወይም የግብርና እንስሳ አለ? | | | 0=አይ | |  |
|  |  |  |  |  | 1=አዎ | |  |
| 6.17 | | በመኖርያ ቤትዎ በአንድ ክፍል ውስጥ ምን ያህል ሰው አለ (በመጠየቅ) …………………………. | | | | | |
| 6.18 | | ከቤተሰብ አባል ውስጥ ሲጋራ የሚጨስ አለ(በመጠየቅ) | 0=አይ | | |  | |
|  |  |  | 1=አዎ | | |  | |

ስለተሳትፎዎ እናመሰግናለን !!
